# Mapping the evidence on the intersection of gender and social determinants of health in health inequality: a scoping review

**DOI:** 10.1186/s12889-025-25525-8

**Published:** 2025-12-17

**Authors:** Babul Hossain, India Pinker, Fatimata Sall, Coralie Dessenne, Maria Ruiz-Castell

**Affiliations:** 1 Socio-Economic & Environmental Health & Health Services (CARES), Research Unit, Luxembourg Institute of Health (LIH), Strassen, Luxembourg; 2 Ageing, Cancer and Disparities Research Unit (ACADI), Luxembourg Institute of Health (LIH), Strassen, Luxembourg; 3 University of Thiès, Thiès, Senegal; 4 Luxembourg Institute of Health (LIH), Strassen, Luxembourg

**Keywords:** Gender inequality and inequity, Health, Social determinants of health, Intersectionality, Regional pattern

## Abstract

**Background:**

For decades, researchers have acknowledged the persistence of gendered health inequalities across the world. Consequently, an intersectional approach has emerged in public health research as an important methodological advancement for studying health inequalities. However, it remains unclear how the intersection of gender with social determinants of health (SDH) shapes health inequalities across different health domains and regions. This review aims to summarise published studies comparing health outcomes between men and women across health domains and regions, framed within the context of SDH and the emerging intersectionality approach.

**Methodology:**

This review followed Arksey and O’Malley’s framework and Levac’s recommendations. We searched electronic databases, including EMBASE, PubMed, PsycINFO, and CINAHL, using predefined inclusion criteria, resulting in 83 research papers selected for data extraction published up to March 15, 2024. Data collection, charting, and review were done using the *Preferred Reporting Items for Systematic Reviews and Meta-Analyses Scoping Review* (PRISMA-ScR) guidelines.

**Results:**

This review highlighted that the intersection of gender with SDH influenced health outcomes and contributed to unequal health care use for both men and women. Findings indicated that the intersection of gender and SDH could lead to a widened gender gap in specific health outcomes (e.g., multimorbidity or mortality risk) within a particular health domain (e.g., physical health). However, this pattern was not consistently observed across other health outcomes within the same domain. The review also found that the factors intersecting with gender to produce health inequality varied by region. In America, race and ethnicity were key factors influencing health inequality between men and women. In Europe, the intersection of economic factors and gender played a major role. In Asia and Africa, gendered health inequality was shaped by the intersection of gender with social position-identity. There were also regional differences in the focus on health domains and outcomes. Across the Americas, Asia, and Europe, physical health was the main health domain of study. Commonly explored outcomes included obesity/BMI in the Americas, cardiovascular health in Asia, and poor self-perceived health in Europe. In Africa, studies mostly looked at the health care domain, especially health-seeking behaviour.

**Conclusions:**

Unequal social positions, identities, economic conditions, and sociodemographic factors contribute to gendered health inequality. Regional patterns reveal the need for more critical and context-sensitive research on health inequalities across genders to inform more nuanced and locally relevant public health strategies.

**Supplementary Information:**

The online version contains supplementary material available at 10.1186/s12889-025-25525-8.

## Background

Over the past few decades, researchers have shown a growing interest in examining the social factors contributing to health inequalities [1–3]. According to the Commission on the Social Determinants of Health (SDH) (2008), health inequality refers to systematic differences in health experiences, shaped by the conditions in which individuals are born, live, work, and die [4]. Among these factors, gender is widely recognized as one of the most significant axes of health inequality [4, 5].

Despite this, public health and epidemiological studies frequently treat gender as a fixed biological attribute - i.e., sex - instead of a dynamic, socially constructed, and context-dependent determinant of health [6]. Sex-based health inequalities originate from biological factors such as genetics, hormones, anatomy, and physiology [7]. In contrast, gendered health inequalities reflect systematic differences in health outcomes, risks, and care use across genders, shaped by socially constructed roles, cultural norms, power relations, and institutional structures [8–10]. These differences often constitute health inequities when they produce unfair and preventable disadvantages [11–13]. Structural factors such as unequal decision-making power, policy gaps that benefit privileged groups, labour market segregation, and unequal access to resources shape intermediate determinants of health [14]. These include education, income, occupation, employment status, housing conditions, immigration status, marital status, social support networks, and access to health information. In turn, these intermediate determinants influence the unequal distribution of chronic disease risk, variations in health care-seeking behaviour, and disparities in access to and utilization of health services across genders [15–17]. Although evaluating structural determinants of health can be complex, examining their intersection with gender can help identify populations at greater risk of poor health outcomes [9, 15, 18].

Gender can have both detrimental and protective effects on health. Influenced by various social determinants, differences in health are observed between men and women [9, 12, 19]. However, these intersections vary across regions. In the Global North (GN), gender is often analysed using theoretical frameworks such as the theory of “gender and power” and the concept of “doing gender” [20, 21]. These frameworks consider gender as intertwined with institutional structures, social roles, and power dynamics, while also simultaneously accounting for socioeconomic differences. In the Global South (GS), these frameworks have only recently been applied. Research here often focuses on context-specific systems of oppression, such as caste or ethnic hierarchies in India or Brazil, or the legacy of colonialism in sub-Saharan Africa, and their intersections with poverty, informal labour, and limited health care access [21, 22]. These regional differences highlight the need for context-sensitive approaches that capture the interplay of multiple determinants in shaping sex and gendered health inequalities and inequities.

### Gender and health- from SDH to intersectionality

Lesley Doyal’s seminal work on the political economy of health, marked a turning point by showing how socio-economic and biomedical factors, particularly gender, shape health outcomes [23]. Doyal highlighted how participation in paid and unpaid work, caregiving, and substance use patterns strongly affect health [23, 24]. Östlin et al. (2001) further emphasized that both biological sex and socially constructed gender jointly influence exposure, vulnerability, and health inequalities [22]. Building on this, researchers began to examine how gendered divisions of labour and caregiving responsibilities contribute to differential health risks and access to health care services between men and women [21, 25, 26].

The field advanced with the WHO’s 2009 SDH framework, which recognized gender as a critical determinant influencing access to resources, opportunities, and power [4, 21, 27]. However, it largely considered gender as an independent factor, overlooking its intersections with other determinants. In response, recent research has adopted intersectionality [28–31], a framework introduced by Kimberlé Crenshaw (1989) to describe the multiple forms of oppression faced by among Black Americans. Intersectionality examines how overlapping identities such as gender, race, class, and migration status, jointly produce inequalities creating complex power dynamics [32–34]. This framework shifts focus from individual factors to broader structural l concerns and supports gender-transformative actions that challenge harmful norms, informing stronger policies and programmes [15, 35].

### Objectives and contributions of this review

There is currently no comprehensive overview of gendered health inequalities that adopts an intersectional perspective across health domains and regions. While recent reviews have called for integrating solution-linked factors and applying gender-sensitive, intersectional approaches at different levels (e.g., state, community, and individual) [15, 30], these remain underexplored. As such, it remains unclear how the intersection of gender and SDH shapes health inequalities across health domains and regions. This review aims to provide an overview of the intersections between gender and SDH across physical and mental health outcomes, self-perceived health, health behaviour, and health care among adults. It also explores and compares these intersections among adults in different regions.

Since most research adopts a binary perspective on gender (men and women), this review follows the same approach to reflect existing literature. However, we acknowledge that gender identity is diverse and that non-binary, transgender, and gender-fluid individuals face unique health challenges and barriers to health care access. These challenges are often rooted in cultural biases, moral beliefs, and societal norms, which differ across regions and historical periods [15]. While the topic is a crucial area of study, addressing these complexities requires a dedicated review beyond the scope of this work.

## Methodology

**Fig. 1:**
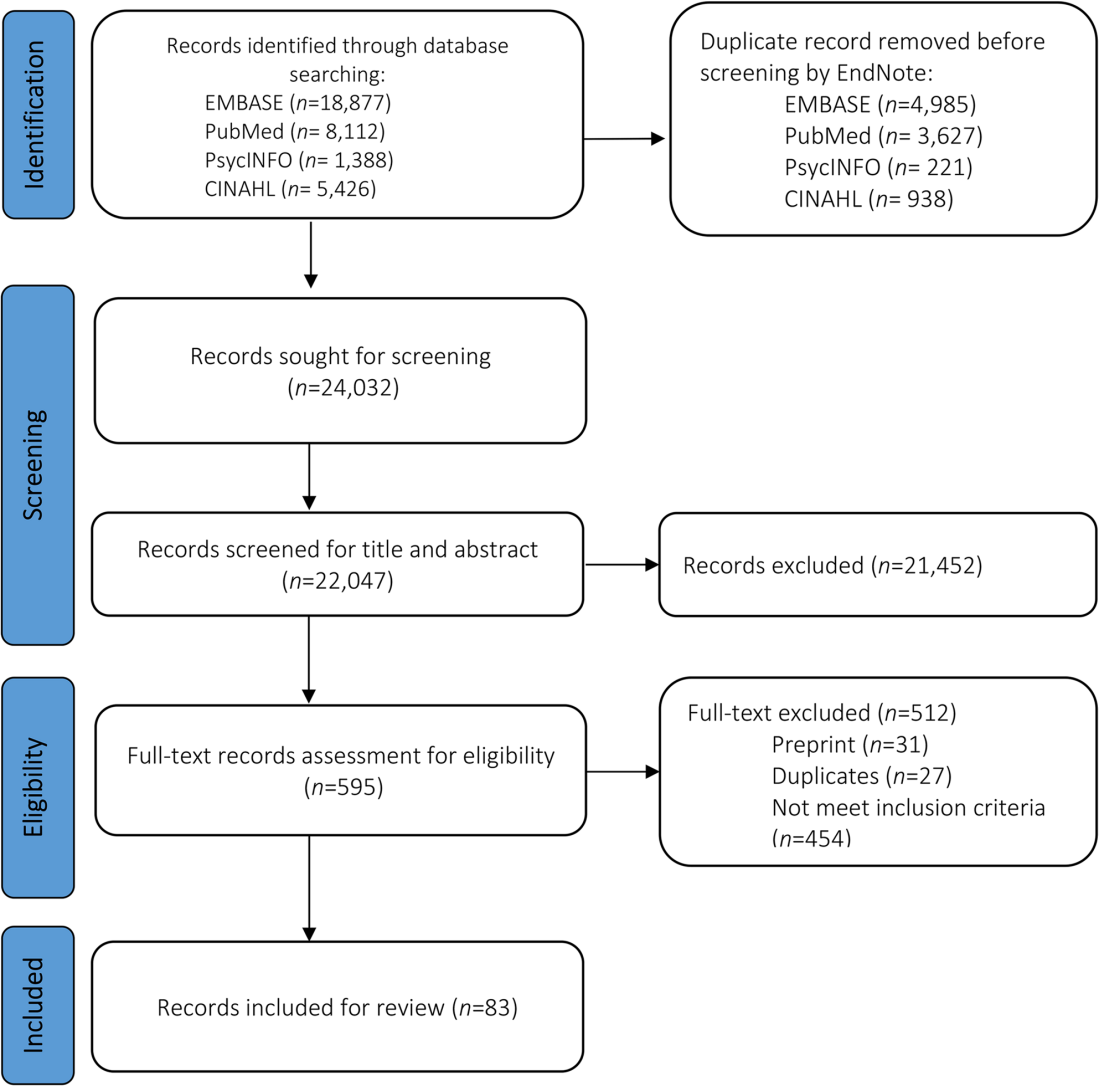
Search process in PRISMA flow diagram

This review followed Arksey and O’Malley’s framework, along with Levac’s recommendations for scoping reviews [36]. Additionally, the *Preferred Reporting Items for Systematic Reviews and Meta-Analyses Scoping Review* (PRISMA-ScR) guidelines were implemented to ensure transparency (see Fig. 1). The protocol for this review was registered on the Open Science Framework platform (https://archive.org/details/osf-registrations-7b32s-v1*).*

### Search strategy

The research strategy focused on how binary gender (men and women) intersects with SDH. These determinants included education, income, employment, marital status, race, ethnicity, immigration, living conditions, exposure to pollution, neighbourhood quality, and risky behaviours. Health outcomes were defined broadly to include as many reportable aspects of health as possible. Given the breadth of the topic and the expected high volume of studies, we limited the search to four electronic databases: EMBASE, PubMed, PsycINFO, and CINAHL. The search included all available studies up to March 15, 2024.

The search strategy was developed with a health-focused research librarian using relevant keywords and Boolean operators (see the Supplementary Table 1). Terms related to both sex and gender were included, as these are often used interchangeably in public health and epidemiological studies. SDH-related terms included socio-economic gradient, social class, education, occupation, income, ethnicity/race, nationality, marital status, environment, pollution, and migration [4]. Terms like disparity, inequality, inequity, difference, gap, and disadvantage were incorporated to capture studies on inequalities, and “health” was included to focus the search on health-related literature. The search employed both Medical Subject Headings (MeSH) and free-text terms.

### Study selection

The eligibility criteria were developed using the Population, Concept, and Context (PCC) framework proposed by Peter et al. (2015), as outlined in Table 1 [37].

**Table 1:** Eligibility criteria based on the population, concept, and context (PCC) framework

Elements	Inclusion criteria	Exclusion criteria
Population	Adults	Children or adolescents <18 years
Concepts	Intersectionality (i.e., studies focused on the intersection between gender and at least one Social Determinant of Health (SDH) characteristic). SDH, including socioeconomic status (*e.g.,* economic factors, educational access, and quality), individual gender/sex, social context (*e.g.,* ethnicity/race, marital status, immigration status), and the built/neighbourhood environment. Binary gender (studies that used the term “sex” to examine gender differences in health).	Studies that analysed only sex/gender without considering intersections with other SDH. Studies focused on a single gender (i.e., not illustrating any differences). Studies centred on the categorisation of self-identifying genders and sexual minorities. Studies with an aim/objective not focused on gender in health (i.e., those that only provided gender-based findings in a post hoc analysis).
Context	Gendered health (*e.g., *health status, health behaviours, and pathways of healthcare usage) in observational studies. Focusing on: Health status: physical health (including ailments and chronic conditions), mental health (i.e., depression, anxiety, stress, and other issues), and mortality. Health behaviours: smoking, alcohol consumption, exercise, diet, and nutritional factors. Healthcare usage: Utilisation of inpatient and outpatient healthcare, along with other treatment methods and therapies.	
Study method	Qualitative Quantitative	Reviews of any kind Case studies/reports
Article type	Original peer-reviewed research articles	Conference Abstracts Abstract only records Book Chapters/Dissertations Opinion pieces/Editorials Protocols
Language	English	
Study period	No restriction	
Geography	No restriction

- Population.

The review focused on adults aged 18 years and above. While health inequalities exist across the life course, the particular vulnerability of children were beyond the scope of this review [38].

- Concepts

Three main concepts were selected to explore the intersections between gender and SDH in health and health care inequalities.

1. *Intersectionality*- This review primarily focused on the intersection of gender and SDH [29]. Rather than a one-dimensional perspective, this approach emphasises the importance of examining multiple, overlapping characteristics to identify individuals most at risk of poor health outcomes. Intersectionality has been applied to understand the discrimination and inequality affecting specific groups. Although some studies did not explicitly adopt an intersectional framework, they were included if they provided relevant evidence on how overlapping social determinants shape gendered health inequalities [28]. As the idea of intersectionality may have existed before Crenshaw, who coined the phrase intersectionality in 1989, the search was not limited by publication date.
2. *SDH*- This framework extended beyond medical perspectives by addressing broader social factors that influence health. SDH are socially produced and maintained through historical, political, and economic processes [39]. As Marmot (2005) argued, determinants such as gender, poverty, and social classes reflect underlying social structures and power relations that shape exposure to health risks [40]. Recognizing these structures helps identify root causes of health inequities, even if fully addressing them is beyond the scope of this review [41].
3. *Binary gender*- While sex and gender are distinct concepts, they are often used interchangeably in public health and epidemiological research. Most social epidemiology and public health studies use the binary concept of gender (men and women) or use sex as a proxy for gender. To reflect this literature, the review included studies focusing on men and women.

- Context

This review aimed to assess gender differences in health outcomes, health behaviours, and health care utilization without restriction to any particular health condition. All potential intersections with SDH that could influence overall health were considered.

No restrictions were placed on publication date or geographical location. However, only articles published in English were included in the review. After removing duplicates, the study selection process followed three stages: (1) Title and abstract screening: two reviewers independently screened records using Rayyan software. All disagreements were resolved by a third reviewer. (2) Full-text screening: The two reviewers independently assessed full texts, with any disagreements resolved through discussion with the third reviewer. (3) Final inclusion: all studies meeting eligibility criteria were included. The PRISMA flowchart details the selection process (see Fig. 1).

### Data extraction

A chart was developed for systematic data extraction, including publication year, country of study, author details, sample size, age groups, health outcomes, and health domains. In this review, a distinction is made between health outcomes, defined as specific diseases or conditions, (e.g. stroke, heart disease, cancer, depression, or hospitalization), and health domains, which denote broader categories that group such conditions. For example, the mental health domain included conditions such as depression, anxiety, stress, and post-traumatic stress disorder (PTSD). Other domains identified in this review were physical health, self-perceived health, health behaviours, and health care use (see Table 3). Finally, additional columns addressed the use of SDH and intersectionality frameworks, and the specific SDH variables examined in relation to gender health inequalities.

#### Collating, summarising, and reporting the results

We provided an overview of the study characteristics, including data source, sample size, age groups, and country of origin. Data were synthesised and organised by health domain: physical health, mental health, general health, health behaviours, and health care (see Table 3). Within each health domain, findings were reported by SDH subcategories grouped into four broad categories. The first was social positions and identities, which included factors such as citizenship, immigration status, marital status, educational level, and living arrangement (with whom individuals live in a household). The second focused on financial circumstances, including an individual’s economic role within the household, their socioeconomic class, overall household or personal income, employment status, financial hardship or poverty, and occupational type. The third was race and ethnicity. Although often treated as social positions and identities [42], race and ethnicity were analysed separately due to the large number of studies focusing on these characteristics. In presenting the findings, we retained the racial and ethnic terms used in the original studies (e.g., Hispanic, Black, African American) to ensure consistency with the primary sources. The fourth included other determinants such as pollution and behavioural risk factors, particularly when considered in relation to gender. Subsequently, this review explored regional patterns by describing intersections between gender and SDH across continents. We also identified additional social determinants that overlapped with SDH and gender, intensifying health inequalities. Finally, data were extracted on the most frequently studied health domains and outcomes across regions to identify global patterns of gendered health inequality.

## Results

Out of the 24,032 records identified, we retrieved and independently reviewed the full texts of 595 articles. During this process, 31 preprints, 27 duplicates, and 454 articles not meeting the inclusion criteria were excluded. Ultimately, 83 records were included in the final review (Fig. 1).

### Study characteristics

Of the 83 studies included, 77 were quantitative, and six were qualitative, with publications spanning from 2003 to 2024 (See Table 2). Among the six qualitative studies, four focused on health care, one on self-perceived health, and one on mental health (see Supplementary Table 2). Study populations ranged in age from 18 years to 80 years and above [43, 44]. Sample sizes varied from 10 to 8,190,990 participants [45, 46]. Most studies were cross-sectional (*n* = 58, 69.87%), followed by longitudinal designs (*n* = 25, 30.12%). Regarding conceptual frameworks, 32.53% (*n* = 27) referenced an intersectional framework, 26.51% (*n* = 22) referred both SDH and intersectionality, 13.25% (*n* = 11) referenced only SDH and 27.71% (*n* = 23) did not explicitly reference either framework. Most studies were conducted in North American countries (*n* = 41; United States (U.S.) and Canada), followed by Europe (*n* = 16), South America (*n* = 8; all from Brazil), Africa (*n* = 4; including Morocco, Kenya, Nigeria, South Africa, Ghana, Gambia, Mali, Guinea, Botswana, and Uganda), China (*n* = 5), and other Asian countries (*n* = 7; India, Iran, Singapore, and South Korea).

**Table 2:** Basic characteristics of the selected studies for scoping review

	Frequency	Percentage (%)
Number of selected studies	83	100
Study method		
Quantitative	77	92.77
Qualitative	6	7.23
Study design		
Cross-sectional	58	69.87
Longitudinal	25	30.12
Conceptual framework		
Social determinants of health	11	13.25
Intersectionality	27	32.53
Both	22	26.51
None	23	27.71
Geographical context		
North America	41	49.40
South America	8	9.64
Europe	16	19.28
Africa	4	4.82
Asia	12	14.46
Cross-continental	2	2.41
Sample size in selected studies (minimum and maximum)	10 and 8,190,990	
Publication year in selected studies (earliest and latest)	2003 and 2024	

Table 3 presents an overview of the health domains examined. Physical health was the most studied domain (*n* = 36, 43.37%), followed by mental health (*n* = 20, 24.10%) health care (*n* = 13, 15.66%), self-perceived health (*n* = 8, 9.63%) and health behaviours (*n* = 6, 7.23%).

**Table 3:** Studies with evidence on the intersection of gender with SDH by health domains

Domain	Health conditions/health care aspects	Frequency (N)	Percentage (%)
Physical health	Cardiovascular disease (CVD), stroke and other heart diseases; cancer; diabetes, hypertension, obesity; multimorbidity or physical health score; COVID; mortality; other (pain/sleep/health markers)	36	43.37
Mental health	Depression/depressive symptoms; anxiety, stress. Post-traumatic stress disorder (PTSD)	20	24.10
Self-perceived health	Self-perceived health status	8	9.63
Health behaviour	Smoking, alcohol and other substance use	6	7.23
Health care	Healthcare use; unmet needs for health care; health-seeking behaviours	13	15.66
Total		83	100

*N =* Number of research articles; % = Percentage

### Summary of the evidence on the intersection of gender and SDH across different health domains

#### Physical health

A total of 36 studies investigated the intersection of gender and SDH in physical health. Of these, seven addressed cardiovascular diseases (CVDs), including stroke, and other heart conditions [43, 47–52]; 10 focused on diabetes, hypertension, and obesity [53–62]; and four examined multi-morbidity or overall physical health scores [63–66]. Additionally, five papers explored mortality [67–71]; one focused on COVID-19 [72], one on cancer [73], two on pulmonary conditions [49, 74], and seven addressed other physical health-related topics like pain, sleep, and other physical health markers [72, 75–79].

- Gender and social positions and identities

Education, marital status, and living arrangements emerged as significant characteristics intersecting with gender in shaping gendered inequalities in physical health. Lower educational attainment was associated with higher rates of overweight/obesity, poor cardiovascular health, and worse pain and sleep quality, with stronger effects observed among women than men across different regions [48, 53, 57, 76, 78]. However, a study from China found that low educational attainment was associated with higher mortality risk in older men than in older women [69]. Regarding marital status, unmarried women in Canada had a higher obesity risk than unmarried men, although this was mitigated by participation in social activities [61]. In contrast, evidence from Sweden, China, and the U.S. indicated that unmarried men showed worse physical health outcomes, including higher frailty [75], and higher mortality risks than married men and women [67, 68, 73].

- Gender and financial circumstances

Physical health inequality between men and women also arose in the context of different financial circumstances. Unemployment increased CVD risk for women in the Netherlands [50] and Sub-Saharan Africa [47]. In Canada, unemployment among immigrant women showed worse biomarkers of chronic disease and malnutrition compared to immigrant men, regardless of their employment status [77]. Lower income among women was linked with poorer physical health scores than counterpart men in Spain [64]. Another study from Spain found that low-income women had the highest risk of diagnosed multimorbidity when compared to high-income men [66]. One study from West Africa also showed similar results, with low-income women experiencing higher rates of multimorbidity than men in the same economic situation [63].

- Gender, race and ethnicity

Thirteen studies showed that the intersection of gender with race and ethnicity shaped physical health [52, 54–57, 60, 62, 65, 70, 71, 74, 79, 80]. In the U.S., Black and Hispanic women faced higher rates of hypertension [55], and obesity [57], greater difficulty with weight loss [54], and poorer physical health scores [65] than White and Black men. However, a study in Brazil found different patterns, with hypertension prevalence highest among Black men, followed by Pardo (Brown) men, and Black women [80]. In the U.S., Native American men had the highest rates of heart-related deaths compared with all other gender, race and ethnic groups [70]. In both the U.S. and Canada, Black adults had the highest prevalence of heart-related problems [71, 74]. Black men in the U.S. also had a greater risk of sleep issues [79] than both White men and Black women.

Furthermore, race and ethnicity overlapped with other factors, contributing to health inequality between men and women. For instance, only among Black men and women, experiences of lifetime discrimination and lower household income during childhood were linked with poor cardiovascular health [52]. Similarly, Black women with low income [60, 62] and low education [56] showed the highest rates of hypertension and obesity compared to other gender, racial/ethnic, and education groups.

- Gender and other factors

Neighbourhood socioeconomic disadvantage (based on neighbourhood-level housing conditions, income, education, and occupational level) was linked to higher BMI in women but not in men [59]. Additionally, air pollution and personality traits like hostility - a long-term predisposition characterized by distrust of others and a tendency to respond with anger - were associated with greater CVD and pulmonary health risks in women than in men [43, 49].

#### Mental health

A total of 20 studies examined the intersection of gender and SDH on mental health, including 13 on depression/depressive symptoms [81–93], two on post-traumatic stress disorder (PTSD) [94, 95], and five on broader mental health outcomes such as general mental health status and anxiety [44, 96–99].

- Gender and social positions and identities 

Consistent with the physical health domain, education, immigration, social support, and social isolation intersected with gender. Lower education was associated with more depressive symptoms in Chinese women but not men [83]. Asian immigrants in the United Kingdom (UK) reported more depressive symptoms than native-born residents, particularly among those whose parents worked in manual occupations [93]. Social support reduced major depressive symptoms in women but not men [92]. However, a study from China found that social isolation worsened cognitive scores in men more than women [44]. Conversely, a qualitative study from the UK reported that women, particularly newly married women, experienced social isolation and poor mental health while living abroad due to caregiving expectations, family pressures, and limited external support [96].

- Gender and financial circumstances

Household financial roles and income levels contributed to mental health inequality between genders. Spanish men who supported their families through manual labor had more depressive symptoms than men who provided financial support through non-manual work [86]. In contrast, the same study found that women working as directors and managers while simultaneously managing both financial provision and household chores had the highest risk of depressive symptoms across all other gender and occupation groups [86]. In Sweden, depressive symptoms among women were associated with job dissatisfaction or insecurity and unmet medical needs, whereas among men, depressive symptoms were linked to material conditions, including limited asset ownership [90]. In contrast, a cross-regional study found no gender inequalities in anxiety related to financial circumstances [97]. Another study from the U.S., however, found that lower family income increased death anxiety among White men compared to their higher-income counterparts [98].

- Gender, race and ethnicity

Gendered mental health inequalities were amplified when intersecting with race and ethnicity. Black women in Brazil had higher rates of common mental disorders compared to White men [99]. Hispanic and White women had more depression than White men in the U.S [85]. Studies from the U.S. found that Hispanic/Latino followed by Asian or Pacific Islander women had higher rates of PTSD compared to White men and women [94, 95].

Furthermore, when gender intersected with race, ethnicity and other SDH, mental health inequalities among men and women were intensified. For instance, studies from the U.S. showed that Black women with lower education levels as well as Black men and Hispanic women with lower income, had a higher prevalence of depression than their White counterparts [81, 84].

- Gender and other factors

Health behaviours also intersected with gender in shaping mental health inequalities between men and women. Exposure to smoking in the U.S [89]., as well as physical inactivity and poor sleep in South Korea [91], were associated with depressive symptoms in women but not men. Elevated BMI was also associated with depression, but the association was observed only in White women [87]. Disability was linked to higher levels of depression among White men and women but not among their African Americans counterparts [82].

#### Self-perceived health

Self-perceived health (SPH) can be a proxy indicator of physical health, psychological health, and disability. A total of eight papers focused on the intersection between gender and other SDH in self-perceived health [100–107].

- Gender, and social positions and identities

Lower educational attainment and immigration status were associated with SPH inequality between men and women in Europe and the U.S [100, 104, 105]. In Barcelona, foreign-born men residents reported the poorest SPH, whereas among women, the poorest SPH was reported by Spanish-born residents who had moved to Barcelona from other regions of Spain [102]. A similar pattern was also observed in the U.S [103].

- Gender, and financial circumstances

Unemployed men reported worse SPH than women [105], whereas women in manual work reported worse SPH than men [101]. Furthermore, women in Sweden with low income had the highest risk of poor SPH when combined with additional characteristics such as immigration status [103]. Similarly, in Spain, women living in less developed regions - defined as a lower level of inequality-adjusted Human Development Index- also faced a higher risk of poor SPH compared to their men counterparts [106].

- Gender, race and ethnicity

In the U.S., SPH had worsened over time among non-Hispanic Black and Hispanic men than White men and women [104]. Additional overlapping social positions further shaped these gender inequalities in SPH. For instance, married Non-Hispanic Black women with higher education showed improvements in self-rated health over time compared to Non-Hispanic Black men [104]. Furthermore, a qualitative study found that gender roles often positioned women as primary care providers, leading them to prioritize the needs of their family members over their own. As a result, women often neglected their own health care, which negatively affected their SPH [107].

#### Health behaviour

Six studies explored gender interactions with different SDH in shaping inequalities in health behaviours. Three of these papers focused on alcohol and drinking problems [108–110], one on smoking [111], and the other two on substance use [112, 113].

- Gender, and social positions and identities

One study in Brazil found a strong link between higher social status (based on schooling and occupation) and an increase in alcohol consumption abuse (ACAb) among men but not women [108].

- Gender and financial circumstances

A study from Brazil indicated that women with higher per capita income (vs. low-income women) and unemployed men (vs. employed men) were more likely to abuse alcohol [109]. In the U.S., men in the lowest income group used marijuana more than women in the lowest income group [112].

- Gender, race and ethnicity

A study from the U.S. on smoking denial found that non-Hispanic Black women were more likely to deny past smoking compared to non-Hispanic White women and Black men [111]. Patterns of substance use disorders (SUDs) also varied by gender and ethnicity. Hispanic women with alcohol use disorders were less likely than White women to experience persistent issues. Similarly, Hispanic men with drug use disorders were less likely to have persistent issues than White men [113]. The same study also reported that Black men with alcohol or drug use disorders showed lower persistent issues than Whites, but those with poly-substance use disorders were more likely to have persistent challenges than Hispanic men [113]. Furthermore, abusive alcohol consumption among Black and Hispanic men was intensified by socioeconomic disadvantage, particularly among those living near the poverty threshold [110].

#### Health care

Thirteen studies examined health care outcomes. Five of the studies focused on the unmet need for health care [114–118] and two focused on mental health care utilization [46, 119]. The remaining three studies focused on hospital stay, diagnosis, and type of health care use [45, 120–124].

- Gender, and social positions and identities

A study conducted in Sweden highlighted that gendered power relations affect access to rehabilitation services. Women reported feeling undervalued and misunderstood, often requiring men’s advocacy to receive equitable treatment compared to their men counterparts [117].

A study from Sweden indicated that highly educated women used more primary care than highly educated men, even after adjusting for diagnosis [122]. In South Africa, COVID-19 disproportionately reduced health care access for women with low education [116]. Furthermore, a qualitative study from Kenya revealed that despite expanded community health services, gendered power relations, social norms, and discrimination influenced health vulnerabilities, affecting women’s health care access [118].

- Gender, and financial circumstances

Income class, financial restraint, and household financial roles influenced gendered inequalities in health care access and use. In Sweden, antidepressant use was highest among middle-income women aged 50–64 without a formal psychiatric diagnosis, and among low-income women aged 35–49 with a formal diagnosis. In contrast, young men, regardless of their income status, showed the lowest use across diagnostic groups [46].

Evidence from India found that unmet health care needs did not differ by gender in higher economic classes, but men in low and middle economic classes had a higher probability of unmet need compared to women in higher classes [115]. Gender-specific norms further intensified these inequalities. One study from India indicated that men’s role as primary decision-makers, combined with the lower prioritization of women’s health due to financial constraints - contributed to delays in seeking tuberculosis (TB) diagnosis and treatment among women [125]. Similarly, gender roles shaped access to timely treatment for both men and women. In Uganda, for example, men delayed treatment for schistosomiasis because they prioritised earning income, whereas women delayed seeking health care due to household and caregiving duties [124].

- Gender, race and ethnicity

Intersections of race/ethnicity and gender in the U.S. influenced cardiovascular care among insured patients, with non-Hispanic Black and Hispanic women receiving fewer recommended treatments and assessments compared to non-Hispanic Whites [120]. In Canada, White men, non-White men, and non-White women accessed mental health services less frequently than White women [119]. In the U.S., Native American men also had higher treatment admissions and overdose deaths for opioids and stimulants than White men and women [45].

Perceptions of health care need also varied by race, ethnicity and gender. A U.S based study found that non-Latino White and African American men were less likely than non-Latino White and African American women to perceive a need for health care [114]. The gendered pattern was not observed among Asian Americans and Afro-Caribbeans.

### Regional patterns in the intersection between gender and SDH

To address our second research objective, we described how gender intersects with other SDH across different regions (see Table 4). We highlighted three key aspects. First, we identified the most frequent SDH categories that intersect with gender. Second, we highlighted additional overlapping factors that intensified inequalities. Lastly, we reported the most commonly studied health domains and health outcomes.

**Table 4:** Mapping gender and SDH intersections in health inequalities: SDH subcategories, common SDH factors, health domains, and specific health outcomes across geography

Countries	Global perspective (Continents)	Main SDH subcategories intersecting with gender in health inequality	Common SDH factors in the intersection of main SDH sub-categories with gender	Top 3 health domains studied	Top 3 specific health outcomes studied
US, Canada	North America (n=41)	Race/ethnicity (n=25)	Race/ethnicity Intersect with income/economic condition (n=4) Race/ethnicity Intersect with education (n=3) Race/ethnicity Intersect with both education and income (n=2)	Physical health (n=20) Mental health (n= 10) Health behaviour(n= 4)	Obesity/BMI (n=6) Depression (n=6) Smoking/alcohol (n=4)
Brazil	South America (n=8)	Race/ethnicity (n=5)	Race/ethnicity Intersect with education (n=2) Race/ethnicity Intersect with income/economic condition (n=2) Race/ethnicity Intersect with skin color (n=1)	Physical health (n=3) Mental health (n= 2) Health behaviour (n= 2)	Cardiovascular health (n=3) Depression (n=2) Alcohol (n=2)
England, Netherlands, Spain, Denmark, Sweden, Austria, France, Germany, Switzerland, Italy, Greece	Europe (n=16)	Financial circumstances (n=9)	Financial circumstances intersect with immigration status (n=4)	Physical health (n= 5) Mental health (n= 4 Self-perceived health (n= 4)	Poor self-perceived health (n=4) Depression (n=4) Healthcare use (n=3)
Kenya, Nigeria, South Africa, Ghana, Gambia, Mali, Guinea, and Botswana	Africa (n=5)	Social positions and identities (n=3)	Social positions and identities intersect with income/economic condition (n=2)	Health care (n=3) Physical health (n=2)	Heath seeking (n=2) Cardiovascular health (n=1) Pain (n=1)
China, India, Iran, Singapore, Japan, and South Korea	Asia (n=12)	Social positions and identities (n=6)	Social positions and identities intersect with income or economic condition (n=2)	Physical health (n=6) Mental health (n=3) Health care (n=2)	Obesity/BMI (n=2) Depression (n=2) Unmet need for healthcare (n=2)

*n =* number of research articles, *SDH = *Social Determinants of Health, Race/ethnicity denotes both racial classifications and ethnic group identities. Financial circumstances refer to various dimensions of economic status, including household wealth, the individual's economic role within the household, socioeconomic class, household or personal income, employment status, financial hardship or poverty, and occupational type. Social position and identity indicators include educational attainment, immigration status, marital status and living arrangements. Common SDH factors in the intersection of main SDH sub-categories with gender mainly highlight studies with two or above intersectional character in gender health inequality analysis

In North America, particularly the U.S. and Canada, race and ethnicity were the most common determinants intersecting with gender to explain health inequalities between men and women (25 out of 41 studies). Evidence from South America (5 of 8 studies) revealed a similar pattern. In both regions, economic factors and education intersected with gender and race/ethnicity, contributing to gendered health inequalities. In Europe, financial circumstances (e.g., household wealth, individual’s economic role within the household, socioeconomic class, household or personal income, employment status, financial hardship or poverty, and occupational type) were the most frequent SDH intersecting with gender in health inequality (9 out of 16 studies). Immigration status emerged as an additional factor intersecting with gender and financial circumstances, further intensifying gendered health inequality. In Asia and Africa, social position and identity (e.g., education, immigration status, marital status, and social network/isolation) were the most relevant factors intersecting with gender. In both regions, economic factors consistently overlapped with these characteristics, thereby contributing to gendered health inequality.

*Health domains and outcomes*: Across the Americas, Europe and Asia, physical health was the most frequently studied domain, followed by mental health. In Africa, however, health care access was the most studied domain, with a focus on health-seeking behaviour. Common health outcomes also varied by region: obesity/BMI in North America and Asia; cardiovascular health in South America; self-perceived health in Europe; and access to care in Africa.

## Discussion

This review examined the evidence on the intersection of gender and SDH, with particular emphasis on the multi-dimensional nature of health inequalities between men and women.

The intersections of gender and SDH are complex and multifaceted but offer an additional perspective on gendered health inequalities. Findings show that intersecting SDH contribute to poor health outcomes and unequal access to health care between men and women. This is consistent with the intersectionality theory, which posits that systems of oppression, such as patriarchy, sexism, racism, and classism, interact to influence lived experiences and health outcomes.

This review found that the intersections of SDH with gender could widen inequalities in specific health outcomes (e.g., multimorbidity) within a particular health domain (e.g., physical health). This pattern, however, was not consistent for all health outcomes within the same domain. For example, low education and being unmarried increased the likelihood of obesity, CVD, and multimorbidity among women [48, 61], while among men these same factors were linked to higher mortality risk [67, 69]. These results suggest that health inequalities cannot be attributed solely to gender or SDH; rather, their intersections create compounded health burdens that manifest differently for men and women [21]. Thus, a nuanced approach must be considered to identify intersectional groups of men and women most vulnerable to poorer health.

Although some studies in this review did not explicitly apply an intersectionality framework, they nonetheless reported overlapping influences of gender and other SDH. While gender is often treated as a control variable in health research, this review shows that its intersections with other determinants actively drive inequalities across health outcomes and domains. This highlights the need for intersectional approaches in public health research and clinical settings. Such approaches conceptualize gender as embedded within broader structural systems - including patriarchy and sexism - that shape access to resources, patterns of discrimination, and ultimately health outcomes [96, 123]. A clear example is the persistent devaluation of women’s health concerns and their underrepresentation in policy priorities, which perpetuate and reinforce gendered health inequalities [118]. This review also identified gender power relations and gender roles, such as caregiving responsibilities, further widened gaps between men and women across health domains [107, 117, 124]. Future research should apply intersectional approaches to explore the structural roots of these inequalities and inform effective interventions.

### Regional patterns

Intersections between gender and SDH varied across regions, emphasizing the need for context-sensitive intersectional approaches.

In North and South America, race and ethnicity were the primary SDH intersecting with gender, highlighting persistent racial and ethnic inequalities [54, 81, 88, 99, 114]. These intersections were further intensified by additional factors such as education, income, and employment status [84, 85, 104, 126]. These findings highlight the need to interpret gendered health inequalities carefully and within broader contexts of ethnocentrism, racism, and social stratification [127].

In Europe, financial circumstances, including income, employment status, job type, and household wealth, were the most frequent determinants intersecting with gender to create health inequalities [50, 72, 86]. These patterns reflect the role of class-based structures, such as job insecurity and lower-income positions, in shaping gendered health inequalities [128].

In Africa and Asia, social positions such as marital status, education, living arrangements, and immigration played a central role in gendered health inequality [43, 67, 115, 116]. In Africa, patriarchal norms and caregiving expectations limited women’s autonomy and access to health care [124]. In Asia, social status, caste systems (separating people at birth into groups based on occupation), and environmental exposures such as air pollution further exacerbated gendered health inequalities [43, 115, 123]. These systems often function within broader patriarchal and hierarchical structures that shape access to education, employment, and health care.

These regional patterns indicate that no universal set of factors explains gendered health inequalities [129]. Instead, region-specific intersections of gender with other SDH must be considered for developing better preventive health strategies to promote global gender equality in health.

Some limitations must be acknowledged. First, this review focused only on men and women, excluding other gender identities - an important gap which future research should address. Second, although this review covered a broad range of health outcomes, it did not delve into specific pathways linking SDH and health. Third, restricting the review to English-language publications may have introduced both linguistic and cultural bias, potentially underrepresenting intersectional patterns in some regions. Another limitation is that some included studies, while offering relevant insights into gender and health, did not explicitly apply an intersectional framework, which may limit the depth of analysis. Finally, regional patterns identified here reflect the distribution of available studies rather than comprehensive coverage of all countries within each region. For instance, all studies from South America were conducted in Brazil, limiting broader generalization. Results should be interpreted with caution and understood within this specific context.

## Conclusion

This scoping review highlights how gendered health inequalities arise from complex intersections of gender with other SDH across regions, health domains, and outcomes. No single factor explained these inequalities; rather, they are influenced by context-specific combinations of race, ethnicity, education, income, immigration, employment, social roles, and environmental conditions, all operating within broader structural systems of patriarchy, sexism, classism, and racism. The review reinforced the importance of adopting intersectional approaches that account for regional and cultural variation to better understand and address health disparities between men and women. Future research should incorporate culturally situated frameworks to identify the structural drivers of inequality to best guide the development of equitable health policies and interventions.

## Supplementary Information

.

Supplementary Material 1.

## Data Availability

No/Not applicable (this manuscript does not report data generation or analysis).
