# Supplementary material for: Mapping the evidence on the intersection of gender and social determinants of health in health inequality: a scoping review
Source: BMC Public Health. 2025 Dec 17;25:4260. doi: 10.1186/s12889-025-25525-8 (PMC12709786; doi:10.1186/s12889-025-25525-8)
Supplement: Supplementary file 1 — Supplementary Material 1. [file 12889_2025_25525_MOESM1_ESM.docx]

| **Supplementary table 1: Search strategy** | | |
| --- | --- | --- |
| **PubMed** | | |
| Search ID# | Key concept | Queries |
| #1 | Health | Health [TW] |
| #2 | Gender | gender [TW] OR Sex [TW] |
| #3 | Inequality | "disparit*"[TW] OR "inequalit*"[TW] OR "inequit*"[TW] OR "difference*"[TW] OR "gap"[TW] OR "disadvantage*"[TW] |
| #4 | Areas of inequality/SDH | "socio-economic status" [TW] OR "socio-economic gradient" [TW] OR "social status"[TW] OR "social class"[TW] OR education[TW] OR occupation[TW] OR income[TW] OR wealth[TW] OR "working status"[TW] OR unemployment[TW] OR wages[TW] OR "labor market"[TW] OR ethnicity[TW] OR nationality[TW] OR "marital status"[TW] OR unmarried[TW] OR environment[TW] OR “life style”[TW] OR “health risky behaviour”[TW] OR pollution[TW] |
| #5 | Intersectionality | Intersection[TW] OR Interaction*[TW] OR intersectional*[TW] OR overlapping[TW] OR multiple[TW] OR combined[TW] |
| #6 | Intersectionality framework | Intersectionality[Mesh] |
| #7 |  | #1 AND #2 AND #3 AND #4 AND (#5 OR #6) |
| **Embase** | | |
| #1 | Health | Health |
| #2 | Gender | gender OR Sex |
| #3 | Inequality | 'disparity' OR 'inequality' OR 'inequity' OR 'difference' OR 'gap' OR 'disadvantaged' |
| #4 | Areas of inequality/SDH | 'socio-economic status' OR 'socio-economic gradient' OR 'social status' OR 'social class' OR education OR occupation OR income OR wealth OR 'working status' OR unemployment OR wages OR 'labor market' OR ethnicity OR nationality OR 'marital status' OR unmarried OR 'life style' OR 'health risky behaviour' OR environment OR pollution |
| #5 | Intersectionality | intersection OR interaction* OR intersectional* OR overlapping OR multiple OR combined |
| #6 | Intersectionality framework | 'intersectionality'/exp |
| #7 |  | #1 AND #2 AND #3 AND #4 AND (#5 OR #6) |
| **PsycINFO** | | |
| #1 | Health | Health.mp. |
| #2 | Gender | (gender or sex).mp. |
| #3 | Inequality | ('disparity' or 'inequality' or 'inequity' or 'difference' or 'gap' or 'disadvantaged').tw. -> disparity or inequality or inequity or difference or gap or disadvantaged.mp. |
| #4 | Areas of inequality/SDH | (socio-economic status' or 'socio-economic gradient' or 'social status' or 'social class' or education or occupation or income or wealth or 'working status' or unemployment or wages or 'labor market' or Race or ethnicity or nationality or 'marital status' or unmarried or environment or pollution).tw.. -> (“socio-economic status” or “socio-economic gradient” or “social status” or “social class” or education or occupation or income or wealth or “working status” or unemployment or wages or “labor market” or Race or ethnicity or nationality or “marital status” or unmarried or environment or pollution).mp. |
| #5 | Intersectionality | (intersection or interaction* or intersectional* or overlapping or multiple or combined).mp. |
| #6 | Intersectionality framework | exp intersectionality/ |
| #7 |  | #1 AND #2 AND #3 AND #4 AND (#5 OR #6) |
| **CINAHL** | | |
| #1 | Health | TX Health |
| #2 | Gender | TX gender or Sex |
| #3 | Inequality | TX disparity or inequality or inequity or difference or gap or disadvantaged |
| #4 | Areas of inequality/SDH | TX “socio-economic status” or “socio-economic gradient” or “social status” or “social class” or education or occupation or income or wealth or “working status” or unemployment or wages or “labor market” or Race or ethnicity or nationality or “marital status” or unmarried or environment or pollution |
| #5 | Intersectionality | TX intersection OR interaction* OR intersectional* OR overlapping OR multiple OR combined |
| #6 | Intersectionality framework | MH intersectionality |
| #7 |  | #1 AND #2 AND #3 AND #4 AND (#5 OR #6) |

| **Supplementary table 2: Characteristics of the selected studies included in the scoping review** | | | | | | | | | | | | | |
| --- | --- | --- | --- | --- | --- | --- | --- | --- | --- | --- | --- | --- | --- |
| **ID** | **Authors** | **Publication year** | **Country of origin** | **Continent** | **Study method** | **Study design** | **Age group** | **Sample**  **size** | **Health**  **outcome** | **Domain of health outcome** | **Framework** | **Drivers of inequality** | **Key findings** |
| 1 | Qin et al. | 2015 | China | Asia | Quantitative | Cross-sectional | ≥ 18 years | 24,845 | Cardiovascular disease (CVD) and stroke | Physical health | None | Air pollution | The link between air pollution on the prevalence of CVD and stroke was observed only for women. |
| 2 | Rosenfield | 2012 | U.S. | North America | Quantitative | Longitudinal | ≥ 15 years | 1,308 | Depressive symptoms | Mental health | Intersectional framework | Race/ethnicity, education | Black women reported fewer symptoms than White women but only among those with education beyond high school. This pattern did not apply to men. |
| 3 | Rueda et al. | 2008 | Europe countries | Europe | Quantitative | Cross-sectional | ≥ 65 years | 9,225 | Poor self-perceived health | Self-perceived health | SDH  framework | Education, living arrangements | Living alone linked with increased risk of poor self- perceived health among women than men. |
| 4 | Schieman and Plickert | 2007 | U.S. | North America | Quantitative | Longitudinal | ≥ 65 years | 898 | Diagnosed depression | Mental health | Intersectional framework | Functional limitations | Increased functional limitation was linked to rising depression over time among White individuals and high-SES women. This pattern was not observed among low-SES African American men and women. |
| 5 | Sabanayagam etal. | 2009 | Singapur | Asia | Quantitative | Cross-sectional | 40-80 years | 2,807 | Overweight/  Obesity | Physical health | None | Education, income | Lower education and income was linked to higher overweight and obesity rates in Malay women, but such a pattern was not observed among Malay men. |
| 6 | Simpson et al. | 2019 | U.S. | North America | Quantitative | Longitudinal | ≥ 18 years | 71,799 | Head and neck cancer | Physical health | None | Marital status | Unmarried men faced a higher risk of death compared to married men and were more likely to be diagnosed at a late stage. Marital status had no impact on women’s survival. |
| 7 | Smits et al. | 2021 | Netherlands | Europe | Quantitative | Longitudinal | ≥ 25 years | 1,688,285 | Out-of-hospital cardiac arrest (OHCA) | Physical health | SDH  framework | Employment | Employment status contributed more to the OHCA burden in women than in men. |
| 8 | Smolen et al | 2018 | Brazil | South America | Quantitative | Cross-sectional | ≥15 years | 3,273 | Common mental disorders (CMD) using the Self-Reporting Questionnaire | Mental health | Both | Race/ethnicity | Black women had a higher prevalence of CMD than White men, while White women had similar rates to White men. |
| 9 | Trevisan | 2020 | Sweden | Europe | Quantitative | Longitudinal | ≥60 years | 1,863 | Frailty | Physical health | SDH  framework | Marital status, age | Single or widow men had higher odds of frailty than married men. Younger divorced women faced greater frailty risk than partnered women. |
| 10 | Zhong et al. | 2022 | U.S. | North America | Quantitative | Cross-sectional | ≥20 years | 53,528 | Weight loss attempts | Physical health | Intersectional framework | Race/ethnicity | Black and Hispanic women were more likely than White women to attempt weight loss. |
| 11 | Arcas et al. | 2012 | Spain | Europe | Quantitative | Cross-sectional | ≥16 years | 29 ,478 | Poor self-perceived health | Self-perceived health | Intersectional framework | Occupation, race/ethnicity | Compared to men, women in manual occupations had higher odds of poor self-reported and mental health, particularly among Black women |
| 12 | Assari et al. | 2017 | U.S. | North America | Quantitative | Longitudinal | ≥60 years | 1,129 | Chronic medical conditions | Physical health | Both | Sleep | Restless sleep in 2001 significantly predicted chronic medical conditions in 2011 among Black women, but not among Black men. |
| 13 | Borrell et al. | 2007 | Spain | Europe | Quantitative | Cross-sectional | ≥16 years | 4,214 | Poor self-perceived health | Self-perceived health | SDH  framework | Migration status, type of job | Foreign men had the poorest health status. Among women, those born in other regions of Spain than Barcelona city—particularly managers, supervisors, or professionals—reported worse health compared to women from Barcelona city. |
| 14 | Fisher et al. | 2008 | U.S. | North America | Quantitative | Longitudinal | ≥18 years | 15,182 | Denial of currently smoking | Health behaviour | None | Age, race/ethnicity | Non-Hispanic Black women were more likely to deny smoking compared to both non-Hispanic White women and non-Hispanic Black men. |
| 15 | Huang et al. | 2022 | China | Asia | Quantitative | Longitudinal | ≥80 years | 2,732 | Cognitive impairment | Mental health | None | Loneliness | Loneliness was linked to a higher risk of cognitive impairment in men, but not in women. |
| 16 | Lee et al. | 2021 | U.S. | North America | Quantitative | Longitudinal | 25–74 years | 7,108 | Cardiovascular health | Physical health | Intersectional framework | Race, SES status | Addressing early-life adversity and midlife SES conditions reduced cardiovascular health risk disparities between White men and Black individuals. |
| 17 | Sultana | 2024 | Canada | North America | Quantitative | Cross-sectional | ≥15 years | 85,619 | Mental health service utilization | Health care | Intersectional framework | Race/ethnicity, income | Unemployed White men with the lowest income were highest too use mental health services than employed White men with highest income. |
| 18 | Villatoro et al. | 2018 | U.S. | North America | Quantitative | Cross-sectional | ≥18 years | 14,906 | Perceived need for mental health care | Health care | Intersectional framework | Race/ethnicity | Men were less likely than women to have a perceived need for mental health care but only among non-Latino whites and African Americans. Among Asian Americans, foreign-born immigrants had a lower perceived need for mental health care than those born in the U.S. |
| 19 | Wemrell et al. | 2021 | U.S. | North America | Quantitative | Cross-sectional | ≥16 years | 133,244 | Poor self-perceived health | Self-perceived health | Both | Migration status, income | Immigrant women with low income had a higher risk of poor self-perceived health compared to native men with high income. |
| 20 | Kanchi et al. | 2018 | U.S. | North America | Quantitative | Cross-sectional | ≥20 years | 1,527 | Diabetes, obesity, hypertension | Physical health | Both | Race/ethnicity | Women had lower cardiovascular risk factors than men. Non-Latino Black women showed higher rates of overweight, obesity, hypertension, and diabetes than non-Latino black men or non-Latino white individuals. |
| 21 | Kang et al. | 2016 | U.S. | North America | Quantitative | Longitudinal | ≥20 years | 13,723 | Mortality | Physical health | SDH  framework | Marital status, living arrangements | Being widowed increased mortality risk, but living with both parents moderated this effect for both men and women, though the effect was stronger for men. |
| 22 | Lamidi | 2022 | U.S. | North America | Quantitative | Longitudinal | 40-59 years | 425,947 | Poor self-perceived health | Self-perceived health | Intersectional framework | Education, race/ethnicity | Married Black women with higher education showed improvements in self-rated health over time compared to White men and women. |
| 23 | Limacher et al. | 2023 | Morocco | Africa | Quantitative | Longitudinal | ≥18 years | 385 | Pain | Physical health | Both | Education, financial hardship | Gender did not moderate the relationship between socioeconomic status and health. However, educational inequalities in general health were more pronounced in women. |
| 24 | Luo et al. | 2019 | China | Asia | Quantitative | Longitudinal | ≥18 years | 29,993 | Depressive symptoms | Mental health | None | Education, marital status | Higher education was associated with lower mental health problems in women, while being widowhood was linked to a higher probability of mental health issues in men. |
| 25 | Mahapatro et al. | 2021 | India | Asia | Quantitative | Cross-sectional | All age groups | 43,240 | Unmet need for health care | Health care | Both | Economic class based on consumption | Unmet healthcare needs did not differ by gender in the higher economic class, but men in low and middle economic classes had a higher probability of unmet needs compared to women in the higher economic class. |
| 26 | Maharlouei et al. | 2020 | U.S. | North America | Quantitative | Cross-sectional | ≥18 years | 65,814 | Good self-perceived health | Self-perceived health | Both | Education, employment, marital status | The effects of higher education and being married strongly associated with better self-perceived health for women, while being employed had a greater association on men. |
| 27 | Peplinski et al. | 2018 | U.S. | North America | Quantitative | Cross-sectional | 45-84 years | 6,814 | Depressive symptoms | Mental health | Intersectional framework | Race/ethnicity, income, type of occupation | Depressive symptoms decreased with an increase in income Black men, and Hispanic women. Depressive symptoms were also decreased for Hispanic men and Black people in management and professional jobs living in neighborhoods with higher socioeconomic status compared to. |
| 28 | Roxburgh et al. | 2014 | U.S. | North America | Quantitative | Cross-sectional | 16–84 years | 13,328 | Depression | Mental health | None | Race/ethnicity | Hispanic women, White men and women reported more foster care experience than Black men and women. However, only Hispanic women had the highest depression followed by White men. |
| 29 | Sia et al. | 2019 | Canada | North America | Quantitative | Cross-sectional | 18-65 years | 2,493 | Chronic inflammation  markers | Physical health | SDH  framework | Unemployment, race/ethnicity, immigration | Unemployed immigrant women had higher chronic inflammation markers than employed native men. |
| 30 | Alimohammadian et al. | 2016 | Iran | Asia | Quantitative | Cross-sectional | 40-75 years | 49,946 | Multimorbidity | Physical health | SDH  framework | Marital status, household wealth | Multimorbidity in men was linked to past smoking, non-Turkmen ethnicity, unmarried, and physical inactivity, while in women, it was associated with lower household wealth. |
| 31 | Filho | 2005 | Brazil | South America | Quantitative | Cross-sectional | ≥20 years | 2,302 | Alcohol Consumption-Abuse (ACAb) | Health behaviour | SDH  framework | Race/ethnicity and working class | Irrespective of race/ethnicity, men with upper-middle working class had higher odds of ACAb than counterparts women. |
| 32 | Santos Alves et al. | 2016 | Brazil | South America | Quantitative | Cross-sectional | ≥ 18 years | 59,402 | Hypertension | Physical health | Intersectional framework | Race/ethnicity, education | Black and brown women had higher hypertension prevalence than white women. Education was inversely related to hypertension only among white and brown women. Among men, there were no differences by race or education. |
| 33 | Arias-de la Torre et al. | 2019 | Spain | Europe | Quantitative | Cross-sectional | 16 to 65 years | 14,247 | Depressive symptoms | Mental health | Both | Main economic role in household, marital status | Poor mental health was linked to being the main earner in men from manual classes. In women from nonmanual classes, poor mental health was associated with being widowed, separated, or divorced and managing household work alone. |
| 34 | Assari et al. | 2019 | U.S. | North America | Quantitative | Longitudinal | ≥50 years | 37,495 | Body mass index | Physical health | Both | Education, income, race/ethnicity | Baseline education and income influenced BMI through physical activity only in White women and White men but it was not observed in Black men and women. |
| 35 | Assari et al. | 2016 | U.S. | North America | Quantitative | Cross-sectional | ≥65 year | 1,074 | Anxiety post-acute myocardial infarction | Mental health | Intersectional framework | Age, income | Age predicted death anxiety only in women. Lower family income was linked to increased death anxiety only among men. |
| 36 | Assari et al. | 2016 | U.S. | North America | Quantitative | Longitudinal | ≥50 years | 37,495 | Body mass index | Physical health | Both | Education, income, race/ethnicity | High education protect White men or women from high BMI. While Income protected from sustained high BMI among Black and White women but not men irrespective of race/ethnicity in both Black and White groups. |
| 37 | Ballering et al. | 2021 | Netherlands | Europe | Quantitative | Cross-sectional | ≥ 18 years | 74,722 | COVID-19 diagnosis | Physical health | None | Working type | Women health care workers were less often tested for COVID-19 than men health care workers. |
| 38 | Carliner et al. | 2017 | U.S. | North America | Quantitative | Cross-sectional | ≥ 18 years | 492,831 | Marijuana use | Health behaviour | None | Income | Men in the lowest income group saw an increase in prevalence of marijuana use from 2007 to 2014, widening the gender gap. |
| 39 | Carter et al. | 2017 | U.S. | North America | Quantitative | Longitudinal | ≥50 years | 19,280 | Depressive symptoms | Mental health | Intersectional framework | BMI, race/ethnicity | Sustained high BMI was associated with increased depressive symptoms in White women but not in White men or Black individuals. No ethnicity group differences were observed in the effects of sustained physical activity on depressive symptoms. |
| 40 | Constante et al. | 2023 | Brazil | South America | Quantitative | Cross-sectional | ≥ 18 years | 87,187 | Major depressive disorder (MDD) | Mental health | Intersectional framework | Education, race/ethnicity | Low-education Black women showed gap in risks for probable and diagnosed MDD compared to highly educated White women, respectively. |
| 41 | Correa-de-Araujo et al. | 2006 | U.S. | North America | Quantitative | Cross-sectional | ≥ 18 years | 35,407 | Acute myocardial infarction and congestive heart failure | Health care | None | Race/ethnicity | Race/ethnicity and gender influenced cardiovascular care among Medicare patients, with Black and Hispanic groups, especially women, receiving fewer recommended treatments and assessments compared to non-Hispanic Whites, |
| 42 | Fan et al. | 2022 | U.S. | North America | Quantitative | Longitudinal | ≥ 20 years | 27,175 | Depressive symptoms | Mental health | None | Tobacco smoke exposure | Active tobacco smoke exposure was linked to depressive symptoms only in women. |
| 43 | Freitas et al. | 2022 | Brazil | South America | Quantitative | Longitudinal | 35-74 years | 13,926 | Score of ideal cardiovascular health (ICH) based on the American Heart Association definition | Physical health | Intersectional framework | Education | The study found a positive interaction between gender and education level, with women who have at least a high school education showing better ideal cardiovascular health scores than counterparts men. |
| 44 | Fuller-Thomson et al. | 2016 | Canada | North America | Quantitative | Cross-sectional | ≥50 years | 12,953 | Chronic Obstructive Pulmonary Disease (COPD) | Physical health | None | Race/ethnicity | Black and White women had the highest prevalence and greater odds of COPD compared to men, even after adjusting for socioeconomic position. Black men had lower odds compare to White men. |
| 45 | Gustafsson et al. | 2016 | Sweden | Europe | Quantitative | Cross-sectional | 16- 84 years | 25,585 | Depressive symptoms | Mental health | Both | Income, unmet need | Depressive symptoms in women were associated with job conditions and unmet medical needs, while in men, they were linked to material conditions. |
| 46 | Abdalla | 2022 | Kenya, Nigeria, and South Africa | Africa | Quantitative | Cross-sectional | ≥15 years | 3,058 | Need to seek health care | Health care | Both | Education | During the pandemic, women—especially those without post-secondary education or financially affected—experienced a significant decline in accessing health care, while men showed no comparable change. |
| 47 | Assari | 2014 | U.S. | North America | Quantitative | Cross-sectional | 30-44 years | 5,191 | Body Mass Index | Physical health | Intersectional framework | Race/ethnicity, anxiety, major depressive disorder | Among Caribbean Black men and African American women, lifetime anxiety was linked to high BMI, while among Caribbean Black women, lifetime major depressive disorder was associated with high BMI. |
| 48 | Assari | 2013 | U.S. | North America | Quantitative | Cross-sectional | 30-44 years | 717 | Sleep Quality | Physical health | None | Education and income | Among women, low education and low income was associated with poor sleep quality but not for men. |
| 49 | Assunção et al. | 2022 | Brazil | South America | Quantitative | Cross-sectional | ≥ 18 years | 33,632 | Hazardous alcohol use | Health behaviour | SDH  framework | Education and employment | Increased gender equality through education and employment over time in European countries was linked to higher alcohol consumption among women, narrowing the gender gap not by reducing men's drinking but by increasing women's. |
| 50 | Hong et al, | 2022 | South Korea | Asia | Quantitative | Cross-sectional | ≥ 60 years | 5,024 | Depressive symptoms | Mental health | None | Sleep, physical inactivity | Physical inactivity and inadequate sleep were linked to depression in women but not in men. |
| 51 | Pal et al. | 2022 | U.S. | North America | Quantitative | Cross-sectional | ≥ 18 years | 1,611,152 | In-patient mortality and stroke risk | Physical health | Intersectional framework | Race/ethnicity | Native American men faced the highest risk of in-hospital mortality. Asian/Pacific Islander women showed the highest stroke risk. |
| 52 | Vaughan et al. | 2019 | U.S. | North America | Quantitative | Cross-sectional | ≥ 35 years | 1,864 | Heart disease death rates | Physical health | Both | Race/ethnicity, age | White women had the lowest rates of heart disease followed by Black women, then white men and  Black men. This pattern was observed for the 45-54 and 55-64 years age groups. While for all age groups < 75 years, White women and White men had the lowest rates of heart diseases than Black women and  Black men. |
| 53 | U. Bender et al. | 2023 | Canada, USa, Switzerland, Italy, Spain, and Australia | Cross-continents | Quantitative | Longitudinal | > 18 years | 2,218 | Hospital length of stay | Health care | None | Age, employment status, country of residence | Age, employment status, and country of residence predicted hospital length of stay for both men and women. |
| 54 | E. Colillas-Malet et al. | 2020 | Spain | Europe | Quantitative | Cross-sectional | 18-65 years | 223 | Physical component score | Physical health | SDH  framework | Income | Women had lower physical health scores (PCS) than men, with age and income influencing PCS. |
| 55 | Q. Deng et al. | 2022 | China | Asia | Quantitative | Cross-sectional | All ages | 378,883 | Out-of-hospital coronary death (OHCD) | Physical health | None | Marital status | Being unmarried or widowed increased OHCD more in women than men, while never being married or divorced increased OHCD in men than women. |
| 56 | R. Dev et al. | 2022 | Ghana, Gambia, Mali, Guinea, and Botswana | Africa | Quantitative | Cross-sectional | ≥18 years | 15,356 | Cardiovascular health index | Physical health | None | Employment status | Unemployment or unpaid worker women showed poorer cardiovascular health (CVH) than counterpart men, with higher rates of hypertension and overweight/obesity. |
| 57 | E.A. Evans et al. | 2017 | U.S. | North America | Quantitative | Longitudinal | ≥18 years | 2,860 | Persistence of substance use disorder (SUD) | Health behaviour | None | Race/ethnicity | Hispanic people were less likely than White people to persist of SUD. Black men showed lower persistence for alcohol or drug use disorder than White men but higher for poly-substance use than Hispanics men. |
| 58 | X. Feng and A. Wilson | 2017 | Australia | Asia | Quantitative | Cross-sectional | ≥15 years | 10,281 | Body mass index | Physical health | None | Neighbourhood socioeconomic status | Women living in more disadvantaged neighbourhoods had higher BMI than men, and this gender gap persisted even after adjusting for health behaviours. |
| 59 | T. gagné and G. Veenstra | 2017 | Canada | North America | Quantitative | Cross-sectional | ≥25 years | 613,909 | Hypertension | Physical health | Intersectional framework | Race/ethnicity, income | Income increases were linked to lower odds of hypertension and diabetes among White women, but not among Black men. |
| 60 | C.R. green et al. | 2009 | U.S. | North America | Quantitative | Cross-sectional | 10-92 years | 4,269 | Sleep disturbance | Physical health | None | Race/ethnicity, age, pain | Black young men with chronic pain were at the highest risk for poor sleep quality and difficulty falling asleep than women of the same group, with the gender gap being highest at younger ages. |
| 61 | Z. Hosseini et al. | 2020 | Canada | North America | Quantitative | Cross-sectional | 45–85 years | 28,238 | Obesity (abdominal and general) | Physical health | None | Marital status | Women with limited social ties—such as being single, socially inactive, or living alone—had higher odds of obesity than women who had, while these associations were weaker or reversed in men. |
| 62 | B. Jackson | 2007 | U.S. | North America | Quantitative | Cross-sectional | 18-30 years | 4,629 | Pulmonary function | Physical health | None | Hostility | Higher hostility was linked to lower pulmonary function across genders. |
| 63 | A.A. Jones et al. | 2023 | U.S. | North America | Quantitative | Cross-sectional | ≥18 years | 583,408 | Opioid and stimulant-attributed treatment admission and deaths | Health care | Intersectional framework | Race/ethnicity | Men had higher treatment admissions and overdose deaths for opioids and stimulants than women, with sharp increases among Native American men compared to Native American men and women. |
| 64 | Y. Luo et al. | 2015 | China | Asia | Quantitative | Longitudinal | ≥65 years | 26,748 | Mortality | Physical health | None | Age, education | Education had a stronger protective effect against mortality for men than for women, particularly among the younger population. |
| 65 | J. McClendon et al. | 2019 | U.S. | North America | Quantitative | Longitudinal | 55-64 years | 1,577 | Self-reported physical health | Physical health | Intersectional framework | Race/ethnicity | Irrespective of race/ethnicity, women’s physical health remained stable over time, while men’s declined. |
| 66 | J. McClendon et al. | 2021 | U.S. | North America | Quantitative | Longitudinal | ≥18 years | 2,343 | Post-traumatic stress disorder (PTSD) | Mental health | Intersectional framework | Race/ethnicity | Black women showed a stronger link between discriminatory stress and increased PTSD severity compared to Black men, while no gender difference was observed among White veterans; Hispanic/Latino men experienced a stronger association than Black men. |
| 67 | D.K. Moser et al. | 2003 | Australia, South Korea, Japan, England, and the U.S. | Cross-continents | Quantitative | Cross-sectional | ≥18 years | 912 | Anxiety after post-acute myocardial infarction | Mental health | None | Age, education, marital status | Women showed higher anxiety levels than men across all countries studied, with no interaction from sociodemographic or clinical factors. |
| 68 | R.H. Perlis et al. | 2021 | U.S. | North America | Quantitative | Cross-sectional | ≥18 years | 73,917 | Major depressive symptoms | Mental health | SDH  framework | Social support | Weekly religious attendance and greater trust in others were linked to lower depression risk, with these protective effects being stronger in women. |
| 69 | Sharifian et al. | 2023 | U.S. | North America | Quantitative | Cross-sectional | ≥18 years | 103 | Post-traumatic stress disorder (PTSD) | Mental health | Intersectional framework | Race/ethnicity | Women were more likely to experience depression and anxiety, but less likely to have PTSD than men. Hispanic/Latino followed by Asian or Pacific Islander women had increased problematic anger than White women and men. |
| 70 | Moreno-Agostino et al. | 2024 | U.K. | Europe | Quantitative | Cross-sectional | 18-30 years | 8,588 | Depressive symptoms | Mental health | Intersectional framework | Immigration status, childhood social class | Asian born men living in the U.K. from a disadvantaged childhood social class had the highest depressive symptoms compared to Native born men and women. |
| 71 | de Silva et al. | 2022 | Brazil | South America | Quantitative | Cross-sectional | 35-74 years | 8,528 | Hypertension | Physical health | Intersectional framework | Race/skin colour | Hypertension incidence was highest among Black men, followed by Brown men and Black women, with White women showing the lowest rates. Even after adjusting for various factors, women from racialized groups (Black and Brown) had higher risks than White women. |
| 72 | Evans et al. | 2018 | U.S. | North America | Quantitative | Cross-sectional | ≥18 years | 32,788 | Body mass index | Physical health | Intersectional framework | Race/ethnicity, income, education | MAIHDA modeling revealed that low-income Black women experienced the highest mean BMI. |
| 73 | Glass et al. | 2017 | U.S. | North America | Quantitative | Longitudinal | ≥18 years | 21,140 | Alcohol consumption | Health behaviour | Both | Race/ethnicity, poverty | The impact of poverty on heavy episodic drinking was more pronounced among Black men and women compared to White men and women |
| 74 | Ljungman et al. | 2022 | Sweden | Europe | Quantitative | Longitudinal | ≥10 years | 8,190,990 | Antidepressant use | Health care | Intersectional framework | Income, immigration status | Among immigrants, antidepressant usage was highest among middle-income women aged 50–64 without a psychiatric diagnosis and low-income women aged 35–49 with a diagnosis. Young men (<35 years) showed the lowest use across the two diagnostic groups. |
| 75 | Moreno-Juste et al. | 2023 | Spain | Europe | Quantitative | Cross-sectional | All age group | 1,086,948 | Multimorbidity | Physical health | Both | Income, immigration status | Low-income migrant women living in urban areas for over 15 years faced the highest risk of diagnosed multimorbidity compared to high-income native living in urban areas. |
| 76 | Nyamande et al. | 2020 | Sweden | Europe | Quantitative | Cross-sectional | 16-84 years | 22,997 | Primary and secondary care utilization | Health care | Intersectional framework | Education | Highly educated women used more primary care than highly educated men. In specialist care use, there was no education and gender intersection. |
| 77 | Pedrós Barnils et al. | 2020 | Spain | Europe | Quantitative | Cross-sectional | 19–88 years | 27,215 | Poor self-perceived health | Self-perceived health | Both | Social class and regional development | Women in manual occupations living in less developed regions, followed by men in similar conditions, reported the poorest self-rated health. |
| 78 | George et al. | 2023 | India | Asia | Qualitative | Cross-sectional | 15-44 years | 140 | Access to Tuberculosis (TB) care and diagnoses | Health care | Both | Financial constraint | Gender-related factors, including male family members’ judgments and lower prioritization of women’s health due to financial constraints, contributed to delays in seeking formal TB diagnosis and treatment. |
| 79 | Wiklund et al., | 2016 | Sweden | Europe | Qualitative | Cross-sectional | 35-65 years | 10 | Access and unmet need to care | Health care | Both | Gender power | Gender influenced access to rehabilitation, with women often feeling undervalued, misunderstood, and requiring advocacy to receive equitable treatment compared to men. |
| 80 | Ssali et al., | 2023 | Uganda | Africa | Qualitative | Cross-sectional | Not specific | 40 FG, 10 interviews | Exposure to schistosomiasis disease, access to treatment | Health care | Both | Economic and domestic roles | Men’s poor economic and leisure activities were linked with the treatment of schistosomiasis, and women’s domestic roles lead to distinct risks and barriers in accessing timely care for schistosomiasis diseases. |
| 81 | Brady et al., | 2019 | Australia | Asia | Qualitative | Cross-sectional | 36-74 | 41 | Life with chronic pain | Self-perceived health | Both | Race/ethnicity, socioeconomic class, and cultural gender roles | Women prioritised family over health, or experienced intensified psychological distress and pain, and experienced the highest poor self-perceived health. |
| 82 | McCollum et al., | 2019 | Kenya | Africa | Qualitative | Cross-sectional | Small subset of 16-18 year age and sample over 18 years | 14 key informant interviews, 50 county-level in-depth interviews, 49 multilevel in-depth interviews, 86 community health in-depth interviews, 14 focus groups, 278 photovoice participants (9 samples of 16-18 years ) | Health and healthcare access | Health  Care | Both | Local culture and norms | Devolution in Kenya aimed to improve equity by shifting health decision-making to the regional level, but gender-based power imbalances persist due to local culture.  Despite policies promoting inclusion, entrenched gender norms and discrimination continue to shape health vulnerabilities and limit the effectiveness of reforms. Community health services had expanded, but gendered barriers to access, acceptability, and use remained particularly for women. |
| 83 | Tang and Pilgrim | 2017 | U.K. | Europe | Qualitative | Cross-sectional | 30-71 years | 22 | Mental health risks and management | Mental health | Intersectional framework | Age, immigration, social isolation | Women, especially brides, faced isolation and heavy caregiving expectations due to cultural and family pressures, often with limited external support due to living abroad, which affected their mental health. Older women also often feel isolated and lonely, even when using community services, due to weak social networks (due to different dialects or distrust of others). |
| Note: SDH= Social determinants of health, SES= Socio-economic status, BMI= Body mass index, MAIHDA= Multilevel analysis of individual heterogeneity and discriminatory accuracy, FG= Focused group | | | | | | | | | | | | | |
